# Assessment of the infectivity of malaria parasites from asymptomatic school children to *Anopheles gambiae* mosquitoes in a high transmission area in Ghana

**DOI:** 10.1038/s41598-025-06844-7

**Published:** 2025-07-02

**Authors:** Mawusi Adepa Mawuli, Linda Eva Amoah, Isaac Kwame Sraku, Dickson Donu, Hamza B. Abagna, Festus K. Acquah, Neils Ben Quashie, Yaw Asare Afrane

**Affiliations:** 1 West African Centre for Cell Biology of Infectious Pathogens (WACCBIP), College of Basic and Applied Sciences, University of Ghana, Legon, Accra, Ghana; 2 Department of Pathology, College of Health Sciences, University of Ghana Medical School, University of Ghana, Korle-Bu, Accra, Ghana; 3 Department of Immunology, Noguchi Memorial Institute for Medical Research College of Health Sciences, University of Ghana, Legon, Accra, Ghana; 4 Centre for Vector-Borne Diseases Research, Department of Microbiology, Medical School College of Health Sciences, University of Ghana, University of Ghana, Korle-Bu, Accra, Ghana; 5 Department of Epidemiology, College of Health Sciences, Noguchi Memorial Institute for Medical Research, University of Ghana, Legon, Accra, Ghana; 6 Centre for Tropical Clinical Pharmacology and Therapeutics University of Ghana Medical School, University of Ghana, Korle-Bu, Accra, Ghana

**Keywords:** Malaria, Membrane feeding, Oocyst, Asymptomatic infection carriers, Ghana, Immunology, Microbiology, Medical research

## Abstract

Asymptomatic carriage of malaria parasite is a major public health issue in Ghana and sub-Saharan Africa. These infections are mostly sub-microscopic and not detected by routine malaria screening methods. Asymptomatic malaria infection carriers serve as an infectious reservoir for malaria transmission. This study assessed the infectivity of asymptomatic children to *Anopheles gambiae* mosquitoes in a high transmission area in Ghana. Ninety-eight (98) healthy children were screened for malaria parasites by microscopy and PCR. Sub-microscopic gametocytes carriage was also determined using RT-PCR. Blood samples from asymptomatic parasite carriers were used in membrane feeding assays of laboratory colonies of *An. gambiae* mosquitoes. Infectivity was assessed by dissection of mosquito midguts and the mosquito infection rate and oocyst densities were recorded. The total participants that were asymptomatic for malaria was 73/98 (74.49%). Malaria parasite prevalence was 13.7% by microscopy and 78.08% by PCR. Sub-microscopic infections accounted for 64.38% (47/73) of the asymptomatic parasite carriers. No gametocytes were detected, however, the *Pf*g377 gene was observed in 33.33% (19/57) of the asymptomatic parasite carriers. Blood from 4 out of 19 asymptomatic carriers, associated with carriage of sub-microscopic gametocytes, were found to be infectious to the *An. gambiae* mosquitoes. The average oocyst density observed was 0.01, with an overall mosquito infection rate of 0.07. This data will be helpful in improving current malaria control efforts in Ghana.

**Supplementary Information:**

The online version contains supplementary material available at 10.1038/s41598-025-06844-7.

## Introduction

Transmission of malaria parasites to *An. gambiae* mosquitoes sustains the transmission cycle. Identification of all infectious reservoirs is relevant to malaria control^1,2^. Asymptomatic infection is characterized by carriage of parasites in the absence of signs and symptoms of malaria, particularly fever^3^. These infections are mostly sub-microscopic^4^ and more frequent in areas of high malaria transmission^5–8^. Sub-microscopic infections with asexual malaria parasites frequently occurs in older children and adults who are asymptomatic, and this has been largely attributed to acquired immunity^9^. Individuals who are asymptomatic would normally not seek treatment, as such, they serve as carriers of gametocytes that could contribute significantly to the transmission of malaria^10–13^.

Gametocyte carriage in asymptomatic malaria infections on the other hand, is common in younger children^14^. The prevalence of gametocytes estimated by microscopy is much lower than by molecular methods such as PCR^15–17^. Across sub-Saharan Africa, gametocyte prevalence diagnosed by PCR ranging from 13% in Gambia^18^49.2% in Ghana^19^ and as high as 91% in Burkina Faso^20^. Immune responses against gametocyte antigens result in the production of anti-gametocyte antibodies^21^. Some of these antibodies have been characterized as having the ability to prevent the completion of the sporogonic cycle of the parasite within the mosquito referred to as transmission blocking. Antibodies to the gametocyte antigens *Pf*s230-CO_LI_ and *Pf*s48/45-6C were among the first to be identified as transmission blocking antibodies. Antibodies to *Pf*s230-CO_LI_ and *Pf*s48/45-6C have been shown to prevent gametocyte /gamete formation (pre fertilization), whiles anti *Pf*s25 and *Pf*s28 antibodies have been shown to prevent ookinete/oocyst formation (post fertilization)^22^. In malaria endemic countries such as Burkina Faso, Ghana and Tanzania, there is the evidence of the presence of antibodies to these pre fertilization antigens^23^. In field settings, naturally acquired transmission-reducing immunity has been shown to reduce infectiousness of gametocytes to female *An. gambiae* mosquitoes^24^. Earlier studies have shown that children are more likely to have anti-gametocyte antibodies as compared to adults^25,26^hence blood samples containing gametocytes from children could be less infectious to *An. gambiae* mosquitoes as compared to blood samples from adults.

The presence of mixed malaria species or different clones of a particular strain have been reported to affect disease outcome^27–29^ and response to treatment^30^. This could also affect the transmission dynamics of malaria. In human malaria infections, mixed infections with *P. falciparum* and *P. malariae* for example, have been shown to result in increased *P. falciparum* gametocytes^27,31^. Also, a comparison of the genotypes of oocysts from a mosquito, with the genotype of parasites from blood in a direct feeding assay revealed that some clones of parasites were not infectious to mosquitoes^32^ whiles others were infectious.

Membrane feeding assays are used to study the infectiousness of gametocytes from natural malarial infections to *An. gambiae* mosquitoes^33^. The source of gametocytes for these assays could either be from cultured gametocytes (standard membrane feeding assay), or from blood samples from individuals with natural *Plasmodium* infections (direct membrane feeding assay). These assays are used for the study of transmission blocking interventions^34,35^ as well as the evaluation of transmission reducing immunity induced by natural malaria infections^36,37^. Here, the infectiousness of gametocytes from asymptomatic children to *An. gambiae* mosquitoes was assessed by direct membrane feeding assays. This will provide information on the role of asymptomatic malaria infections to the transmission of malaria, to assist in decisions pertaining to control of infectious reservoirs of malaria in Ghana.

## Results

### Demographic and clinical characteristics of asymptomatic study participants

Overall, 98 participants were recruited and screened for malaria by RDT, microscopy and PCR. Twenty-five out of the 98 cases were RDT positive, but negative for microscopy and PCR, and were not characterized as negative. The rest (73) of the cases were positive for either RDT, or microscopy, and PCR, and were described as asymptomatic for malaria. The demographic and clinical characteristics of these asymptomatic individuals are represented in Table 1. Out of the 73 asymptomatic cases, 10 (13.70%) were positive for *Plasmodium* infection by microscopy. Parasite density (Geomean (95% CI)) amongst these 10 individuals who were positive for microscopy was 2560.72 (1383.29–6903.39). PCR analysis showed that 57 out of the 73 (78.08%) asymptomatic participants had malaria infections. All cases that were microscopy positive were PCR positive. Individuals who were negative by microscopy, but positive by PCR, were described as having sub microscopic densities of parasites. This constituted 64.38% (47/73) of the total number of asymptomatic individuals. *Plasmodium* species identification indicated that 82.46% (47/57) of the PCR positive cases were mono infection with *P. falciparum* only. The rest of the infections were mixed, with 10.53% (6/57) having *P. falciparum/P. malariae* and 7.02% (4/57) having *P. falciparum/P. ovale* infections. No *P. vivax* was identified in any of the infections.

### Gametocyte carriage in asymptomatic study participants

There were no gametocytes from the microscopic examination of Giemsa-stained thick blood smears. Reverse-transcriptase PCR followed by gel electrophoresis, however showed amplification of the *Pf*g377 gene in 33.33% (19/57) of the study participants. All gametocytes were clonal with observed alleles being either 300 or 350 [base pairs (bp)]. Majority (68.42%, 13/19) were of the 300 bp allele (Supplementary Fig. 1).

**Table 1:** Demographic and clinical characteristics of asymptomatic study participants.

Characteristic	Value
Median age (years) (IQR)	12 (3)
Sex ratio (Male: Female)	40:33:00
Axillary temperature (mean+/- SEM)	36.39+/- 0.05
Haemoglobin concentration (g/dL) (mean+/−SEM)	11.32+/− 0.13
Total WBC	6.15+/- 0.39
Platelet count	229.37+/- 8.87
Total positive for malaria by RDT/microscopy/PCR	73 (74.49)
No. positive by microscopy	10 (13.70)
No. positive by PCR	57 (78.08)
No. with sub microscopic infections	47 (64.38)
No with gametocyte (microscopy)	0
Gametocyte by RT-PCR (*Pf*g377)	19/57 (33.33)
*Plasmodium* species identification	
*P. falciparum* mono infection	47/57 (82.46)
*P. falciparum/P. malariae*	6/57 (10.53)
*P. falciparum/P. ovale*	4/57 (7.02)
PD (p/µl) Geomean (95%ci)	2560.75 (1383.29-6903.39)
PD (p/µl) Min–max	960–10,320

p/ul- parasite per microlitre of blood; CI- Confidence interval; IQR- Inter quartile range; g/dL – grammes per decilitre of blood; WBC-white blood cell count; SEM- Standard error of the mean.

### Mosquito infections

A total of 73 feeding experiments were carried out using blood samples from the asymptomatic study participants. In total, 2,121 female *An. gambiae* mosquitoes were fed with blood. Blood feeding rate was 56.62% (1201/2121). The total number of mosquitoes that were dissected was eight hundred and sixty-two. Infections were observed only in cases with sub microscopic gametocyte infections. Blood samples from all cases with microscopic asexual parasites did not result in mosquito infections. Out of the 19 participants who had sub microscopic gametocytes, blood samples from 21.05% (4/19) of them were infectious to the *An. gambiae* mosquitoes. Three of the participants whose blood resulted in infections were between the ages of 11–15 years, while the other participant with positive mosquito infections was greater than 15 years (Table 2). The overall prevalence of human to mosquito infectivity amongst the asymptomatic participants was 5.48% (4/73). Considering each experiment individually, the number of oocysts observed in a midgut, ranged from 1 to 13. The oocyst density for the lowest and highest oocyst counts were 0.03 and 1.86 oocyst per mid-gut, respectively. The total number of oocysts observed within infected midguts were 28, with an average oocyst density of 0.01 (Table 2).

**Table 2:** Summary of mosquito infection studies.

Age group (years)	No. of participants blood fed to mosquitoes	No. infective to mosquitoes	No. of mosquitoes dissected	No. of infected mosquitoes with oocyst	No. of oocyst	Infection rate	Oocyst density
< 5	1	0	9	0	0	0	0
5–10	25	0	230	0	0	0	0
11–15	41	3	560	10	19	0.02	0.03
> 15	6	1	63	3	9	0.05	0.14
Total	73	0	862	13	28		

### Sero prevalence of antibodies to *Pfs*230-C0_Ll_ and *Pfs*48/45-6C in asymptomatic study participants

The median antibody concentration, (Inter quartile range, QR) (1,866.29, (1,186.85) for *Pf*s230-CO_LI_ was significantly higher (Mann Whitney U test, 0.00), than that for *Pf*s48/45-6C (1,217.42, (IQR) 1,073.67). Seroprevalence of antibodies to *Pfs*230-C0_Ll_ and *Pfs*48/45-6C were 37.0% and 46.60% respectively.

### Antibody responses and mosquito infections

**Fig. 1:**
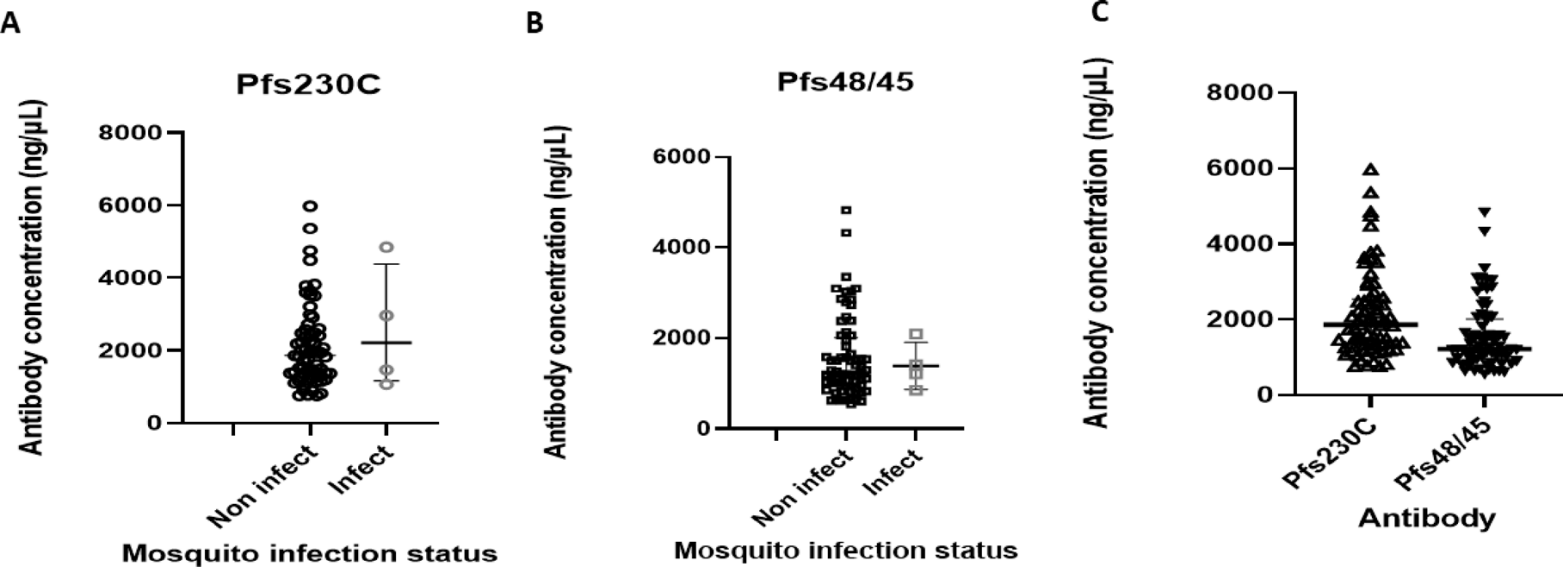
Antibody titers against Pfs230C (**A**) and Pfs48/45 (**B**) amongst study participants whose blood samples resulted in mosquito infections (Infect), as well as those which did not result in mosquito infections (Non infect), and antibody titers for Pfs230C and Pfs48/45 for asymptomatic cases (**C**).

Antibody responses to *Pf*s230C and *Pf*s48/45 varied in the 4 cases that resulted in mosquito infections. Two out of the 4 cases had antibody responses to both antigens, one case had antibody response to *Pf*s23C only; and the other case had no response to either of the 2 antigens. The median (Inter quartile range (IQR)) of antibody to *Pf*s230C amongst study participants whose blood samples resulted in mosquito infections was 1509.64 (1377.43). The median (IQR) of antibody to *Pf*s230C amongst participants whose blood samples did not result in mosquito infections was 1941.30 (1186.85). With regards to antibodies to *Pf*s48/45, the median (IQR) for cases that infected and did not infect the *An. gambiae* mosquitoes are 1364.73(2245.02), and 1217.42(940.7) respectively. Figure 1 shows a scatter plot of antibodies against *Pf*s230C and Pfs48/45 among asymptomatic study participants with and without resultant mosquito infections.

Figure 1. Antibody titers against *Pfs230C* (A) and *Pfs48/45* (B) amongst study participants whose blood samples resulted in mosquito infections (Infect), as well as those which did not result in mosquito infections (Non infect), and antibody titers for *Pfs230C* and *Pfs48/45* for asymptomatic cases (C).

## Discussion

Carriers of asymptomatic malaria infections could habour gametocytes that could be infective to *Anopheles* mosquitoes^7,8,14^. Identification of such individuals for appropriate treatment is crucial for the interruption of the malaria transmission cycle^1,2^. In this study, asymptomatic school children were selected for direct membrane feeding assay to determine the infectiousness of their gametocytes to laboratory reared *An. gambiae* mosquitoes. Consistent with previous studies conducted in the same community^38^asymptomatic malaria is a persistent issue and is associated with a high prevalence of sub-microscopic infections in children. Carriage of gametocytes by children within the community has however been reported to be low. In previous studies, prevalence of gametocytes by microscopy was reported to be between 0 and 3.10% for off peak and peak seasons respectively^19^. There were no gametocytes recorded by microscopy in this study where sample collection was done within the peak season. Consistent with earlier studies within the Southern part of Ghana which include the site for this study, sub-microscopic gametocytes is frequently reported ranging from 20 to 49.20%^8,19^.

There are varying reports on the infectivity of microscopy detected gametocytes, and sub- microscopic gametocytes to *Anopheles* mosquitoes. Presence of microscopy detecting gametocytes or sub- microscopic gametocytes alone does not guarantee the infectivity of gametocytes to *Anopheles* mosquitoes. Studies conducted in Burkina Faso reported similar mosquito infection rates for blood samples collected from study participants who were gametocyte positive by microscopy and those who had sub- microscopic gametocytes^39^. Infectivity of sub- microscopic gametocytes from symptomatic children to *Anopheles* mosquitoes have also been frequently reported in Kenya^40,41^. A similar study conducted in South East Asia (Cambodia) reported a mosquito infection of 6.25% (3/48) in 44% of individuals who carried sub-microscopic gametocyte in symptomatic cases^42^. In this same study, asymptomatic individuals on the other hand had a gametocyte prevalence of 12.60% but these gametocytes were not infectious. The implications of this is that sub-microscopic gametocytes in asymptomatic individuals were of very low densities hence could not result in mosquito infectivity. This study reports a high prevalence of sub-microscopic gametocytes with low infectivity to *Anopheles* mosquitoes.

Other factors, aside the presence of gametocytes, could influence the infectivity of gametocytes to the *An. gambiae* mosquitoes. These factors include; presence of transmission blocking antibodies^22,43–45^innate immune response to gametocytes/gametes within the mosquito^46–48^and polymorphisms in some proteins of the mosquito such as the fibrinogen-related protein − 1 (FREMP-1)^49^ and the thioester binding protein-1 (TEP 1)^50^. The presence of transmission blocking antibodies (anti *Pf*s230 and *Pf*s6C) did not prevent mosquito infections in this study; two out of the 4 participants whose blood samples resulted in mosquito infections were seropositive for *Pf*s230 and *Pf*s6C. This could possibly be as a result of the presence of other factors that can affect the infectivity of gametocytes as stated earlier. Also, even though the cases were seropositive, the concentrations of these antibodies might not be high enough to prevent transmission of parasites to the *An. gambiae* mosquitoes. Unexpectedly, the highest oocyst count was observed in those cases that were seropositive for both *Pf*s230C and *Pf*s6C. Generally, the presence of these transmission blocking antibodies has been shown to greatly reduce mosquito infections in both field and laboratory assays^22,51,52^. There are instances, however, when this transmission-blocking activity has been observed either with *Pfs230* only^43^ or *Pfs48/45* only^44,53^. It would be expected though, that the combined effect of these two antibodies would lead to greater transmission blocking activity.

The seroprevalence of antibodies to *Pfs230* and *Pfs48/45* reported in this study were low. This could be as a result of the presence of sub-patent gametocytes which did not elicit a strong immune response. Sub- microscopic gametocytes seemed not to elicit adequate immune responses to the gametocytes because of their very low numbers. Effective immune responses to gametocytes have been reported to be dependent on ongoing infections with high density gametocyte infections rather than low density or sub-microscopic infections^36^. The high carriage of asymptomatic malaria infections in the study participants highlights the need to expand control efforts to target asymptomatic malaria carriers to help in reducing malaria transmission.

This study had several limitations. First, Kisumu strain of *Anopheles gambiae* is very susceptible to *Plasmodium* infections due to its adaptation in the insectary. However, it was logistically challenging to breed wild-caught mosquitoes from Obom and adapt them to feed on the membrane feeder. Besides several studies have used the Kisumu strain for infection experiments. Second, a quantitative measure of the sub microscopic gametocytes will have provided additional information that could justify the low infections and also variability in oocyst count. Third, a higher number of feeds would have also improved the precision of this study.

## Methods

### Study site

The study site was Obom (5.6335 N″, − 1.762W″), a community in the Ga South municipality of the Greater Accra region of Ghana. Malaria transmission in Obom is perennial with high transmission^54^. The average rainfall is 790 mm along the coast and 1,270 mm in the extreme North. August is the coolest month with a temperature of 25.1 °C, whiles February and March have a temperature of 28.4 °C. Relative humidity is about 75% in February and March^8,19,38,54^.

### Study design and population

The study was prospective involving purposive sampling and screening of healthy school children between 6 and 17 years for asymptomatic malaria parasite infections including gametocyte carriage. Blood samples from these asymptomatic children were used in a direct membrane feeding assay, to assess the infectiousness of gametocytes from the individuals to *An.gambiae* mosquitoes. Mosquito infections were determined by microscopic examination of mercurochrome-stained mosquito midgut on day 7 after blood feeds. Oocyst density and mosquito infection rate were recorded.

### Asymptomatic case definition

Participants were described as asymptomatic for malaria if they were positive for malaria by RDT, microscopy, or PCR, had an axillary temperature of ≤ 37 °C and did not present with any other signs and symptoms of malaria. The RDT was done using the One Step Malaria (HRP)-II (P.f) and (pLDH) (P.f) Antigen Rapid Test kit from SD BioLine. The kit detects the HRP II and (pLDH) from *Plasmodium falciparum* in human whole blood. Conventional PCR, employing the nested method was used for the determination of malaria parasites, all participants were screened using RDT, microscopy, and PCR.

### Inclusion/exclusion criteria

Participants were included if they qualified as a case of asymptomatic malaria infection carrier and provided assent and or consent. Exclusion involved cases of symptomatic malaria or lack of consent/assent.

### Sample size determination

Sample size was calculated to be a minimum of 54 feeds with the assumption of a 60% successful feeding rate and a 20% infection rate^55^ with samples from asymptomatic individuals.

### Participant recruitment and sample collection

Recruitment of asymptomatic study participants started in May until the end of November 2018, a period which coincided with the second rainfall season. Written informed consent was obtained from adult participants as well as parents/guardians of minors before recruitment into the study. Venous blood samples were drawn using a butterfly needle and following the appropriate protocol for venipuncture. One milliliter of venous blood was drawn each, into a heparin tube and EDTA tube. The heparin preserved samples were kept in a thermos flask at 37 °C, and immediately used for the direct membrane feeding assay. Samples preserved in EDTA were used as follows; malaria rapid diagnostic test, thick and thin blood smears for microscopy. The rest of the EDTA preserved samples were separated into plasma and packed red cells. Subsequently, 100 µl of packed red cells was immediately preserved in 500 µl of trizol, and, 100 µl of packed red cells was also preserved in 400 µl of DNA lyses buffer. These were then transported on ice from the field to the laboratory where plasma samples were preserved at -80 °C. Samples preserved in trizol, DNA lyses buffer, and the rest of the packed red cells were preserved at -20 °C for later laboratory experiments.

### Rapid malaria testing

The One Step Malaria (HRP)-II (P.f) and (pLDH) (P.f) Antigen Rapid Test kit from SD BioLine, which detects the HRP II and (pLDH) from *Plasmodium falciparum* in human whole blood was used as one of the screening test for malaria. A test was recorded as positive if any of the test lines showed in addition to the control line. A test was recorded as invalid if the control line failed to show.

### Giemsa staining of blood smears and malaria parasite count by microscopy

Parasite density (PD) per microlitre (µL) of blood was determined by microscopic examination of giemsa stained thick blood smears under oil immersion. Asexual parasites were counted per 200 white blood cells, and gametocytes were counted per 500 white blood cells. A white cell count of 8,000/µL was used to determine parasite density. A smear was reported as negative if there were no malaria parasites seen after 100 high power fields were observed. High discrepancies between 2 parasite counts warranted a third count by third highly experienced microscopist. The average of the 2 closest counts were then taken as the parasite count.

### Membrane feeding assay and mosquito midgut dissections

Insectary colonized Kisumu strain of *An. gambiae* mosquitoes obtained from the Department of Medical Microbiology, University of Ghana Medical School and the Vestergaard Insectary of the NMIMR were used for the mosquito infectivity studies. This strain has been used for infections studies in the past (ref) For the membrane feeding assays 3–5 day old female *An. gambiae* mosquites were used. Prior to blood feeds, mosquitoes were starved for 5 h. About 60 mosquitoes per paper cup were fed with whole blood using a glass membrane feeder (Hemotek) with a 14 mm diameter. The feeder was equipped with a water jacket connected to a circulating water bath maintained at 37 °C. The *An. gambiae* mosquitoes were allowed to feed for 10 min on 200 µl of heparinized blood kept at 37 °C in a thermos flask. Unfed mosquitoes were aspirated from the cup into an empty cup and discarded into a biohazard bin after spraying them with absolute ethanol. The number of fed mosquitoes were recorded. These were transported back to the insectary following the precautions necessary for transport of mosquitoes, and were maintained on 10% sugar solution under the following room conditions: temperature of 27 °C+/− 2 °C, humidity of 76+/− 5 g kg^−1^, and a 12 h day/12 h night light schedule^56^.

### Dissection of fed *An. gambiae* mosquitoes

Seven days after the *An. gambiae* mosquitoes were fed, surviving mosquitoes were dissected on a microscope slide in phosphate buffered saline under a dissection microscope. Prior to dissection, mosquitoes were anaesthetized by chloroform and were transferred onto cotton wool soaked with normal saline in a petri dish and covered to prevent any escape. The midgut of the mosquito was carefully pulled from the posterior end of the mosquito while the thorax was held firmly by another forceps, transferred onto fresh slides and stained with 0.05% mercurochrome for 25–30 min. Stained slides were covered with a cover slip and observed under the light microscope (X10 and X40 magnification) for the presence of oocysts. The number of individuals whose blood was infective to at least 1 mosquito, the proportion of infected mosquitoes out of the total number of mosquitoes dissected, as well as the number of oocysts per infected mosquito (oocyst density) were recorded.

### *Plasmodium falciparum* species identification

Genomic DNA was extracted from samples preserved in DNA lyses buffer using the Quick-DNA miniprep kit (cath no. D3025) from Zymo Research, California, following instructions in the manual. The concentrations and purity of the eluted DNA was checked using the nanodrop. The DNA samples were kept frozen at -20 °C until use.

Nested PCR was used for *Plasmodium* species identification using genus and species-specific primers that target the *18 S rRNA* gene^57^. In the nest 1 reaction, 5 µL of DNA template was used and 0.5 µL of nest 1 product was used for the nest 2 reaction. All reactions were carried in a volume of 15 µL. For both nest 1 and nest 2 reactions, the master mix contained 167 nM dNTP, 2.5 nM MgCl_2,_ 80 nM of each primer and 1U of One Taq polymerase. The reaction cycling conditions were: initial denaturation for 5 min at 94 °C, followed by 35 cycles of 30 s denaturation; annealing for 1 min at 55 °C (58 °C for nest 2), and extension for 1 min 68 °C; and a final extension for 5 min at 68 °C. For a positive control, a 3D7 *Plasmodium falciparum* stain was used, while a DNA no-template control was used as a negative control.

### RNA extraction and conversion to cDNA

Ribonucleic acid was extracted from trizol preserved whole blood samples using the Direct zol™ RNA Miniprep plus (Zymo, USA) following manufacturer’s protocol. Deoxyribonucleic acid was removed by DNase 1. Elution of RNA was done with 20 uL of DNAse/RNAse free water into DNA/RNA free tubes. Quality and concentrations of RNA was checked with a nanodrop at 260/280 absorbance. A ratio of 2 or more was accepted. In order to check for contamination with genomic DNA (gDNA). the human IL-10 gene was amplified using the extracted RNA samples. Complementary DNA (cDNA) was then synthesized using a ProtoScipt II first strand cDNA synthesis kit (New England BioLabs Inc, USA) following instructions in the manual with slight modifications. Following this, the gametocyte specific gene *Pf*g377 was amplified in a nested reverse transcriptase PCR using specific primers in a protocol earlier described by Menegon and colleagues in 2000^58^. Complementary DNA preparation from gametocytes of the *P. falciparum* NF54 was used as positive controls.

### Detection and quantification of IgG antibodies to *Pfs*230-C0_Ll_ and *Pfs*48/45-6C

The *Pf*s230_LI_ and *Pf*s48/45-6 C proteins, which are sexual stage antigens, and produced using a *Lactococcus lactis* expression system was used in an indirect ELISA to quantify natural immune responses to these antigens as previously described^59^. These antigens, diluted in bicarbonate buffer at 1.0 µg/ml and 0.5 µg/ml respectively, were used to coat NUNC Maxisorp 96-well ELISA plates and incubated overnight at 4 °C. Plates were then blocked with 150 µL of 3% skimmed milk in phosphate buffered saline (PBS) supplemented with 0.05% Tween 20 (PBST) for an hour. Following this, patient serum (1 in 200 dilution), standard and negative and positive controls were incubated. A rabbit polyclonal anti- human immunoglobulin G (IgG) - horse raddish peroxidase (HRP) was used as a conjugate at a 1/3,000 dilution. Colour was developed with 3, 3’, 5, 5’ tetramethylbenzidine. The optical densities (OD) of the plasma samples were transformed into IgG concentrations (ng/µL) based on the regression curve obtained from dilutions of the PB055 (standard) using the ADAMSEL software (Ed Remarque). Positive and negative controls was used on each ELISA plate. These controls consisted of plasma from individuals who have been previously^19^ described as seronegative and seropositive for the antigens. A recombinant IgG (BPO55, The Binding Site) was used as a Standard for the measurements of IgG. Cut offs for antibody responses were obtained by the formula; (Average + 2 Standard deviation) of concentrations of pooled negative control serum.

### Data analysis

Data was entered and analyzed using the statistical package for social sciences (SPSS) version 24. Summaries of data were presented using tables and graphs. Age of participants was reported using median and interquartile ranges. Axillary temperature and hemoglobin results were summarized using means and standard error of means. Ratios were used to describe number of participants by gender. The median and interquartile range was reported for antibody concentrations of *Pfs*230-C0_Ll_ and *Pfs*48/45-6C, and the Mann Whitney U test was used to compare the differences in median antibody concentrations. A probability value of less than 0.05 was considered as statistically significant.

## Electronic supplementary material

Below is the link to the electronic supplementary material.

Supplementary Material 1

## Data Availability

The datasets generated and/or analysed during this study are available from the corresponding authors on reasonable request.

## References

[1] Lin Ouédraogo, A. et al. Dynamics of the human infectious reservoir for malaria determined by mosquito feeding assays and ultrasensitive malaria diagnosis in Burkina Faso. *J. Infect. Dis.* **213**, 90–99. 10.1093/infdis/jiv370 (2015). 26142435 10.1093/infdis/jiv370

[2] Li, J., Docile, H. J., Fisher, D., Pronyuk, K. & Zhao, L. Current status of malaria control and elimination in Africa: Epidemiology, diagnosis, treatment, progress and challenges. *J. Epidemiol. Glob. Health* **14**, 561–579. 10.1007/s44197-024-00228-2 (2024). 10.1007/s44197-024-00228-2 PMC11442732 38656731

[3] Laishram, D. D. et al. The complexities of malaria disease manifestations with a focus on asymptomatic malaria. *Malar. J.* **11**, 29. 10.1186/1475-2875-11-29 (2012). 22289302 10.1186/1475-2875-11-29 PMC3342920

[4] Sattabongkot, J. et al. Prevalence of asymptomatic Plasmodium infections with sub-microscopic parasite densities in the northwestern border of Thailand: A potential threat to malaria elimination. *Malar. J.* **17**, 329. 10.1186/s12936-018-2476-1 (2018). 30208895 10.1186/s12936-018-2476-1 PMC6134695

[5] Iwagami, M. et al. The detection of cryptic Plasmodium infection among villagers in Attapeu province, Lao PDR. *PLoS Negl. Trop. Dis.* **11**, e0006148–e0006148. 10.1371/journal.pntd.0006148 (2017). 29261647 10.1371/journal.pntd.0006148 PMC5754130

[6] Tun, S. T. T. et al. Towards malaria elimination in Savannakhet, Lao PDR: Mathematical modelling driven strategy design. *Malar. J.* **16**, 483–483. 10.1186/s12936-017-2130-3 (2017). 29183370 10.1186/s12936-017-2130-3 PMC5706414

[7] Ndong, I. C. et al. Prevalence of asymptomatic malaria parasitaemia following mass testing and treatment in Pakro sub-district of Ghana. *BMC Public. Health*. **19**, 1622. 10.1186/s12889-019-7986-4 (2019). 31795981 10.1186/s12889-019-7986-4 PMC6889629

[8] Ayanful-Torgby, R., Quashie, N. B., Boampong, J. N., Williamson, K. C. & Amoah, L. E. Seasonal variations in *Plasmodium falciparum* parasite prevalence assessed by varying diagnostic tests in asymptomatic children in southern Ghana. *PLoS One*. 10.1371/journal.pone.0199172 (2018). 29906275 10.1371/journal.pone.0199172 PMC6003688

[9] Smith, T., Felger, I., Tanner, M. & Beck, H. P. Premunition in *Plasmodium falciparum* infection: Insights from the epidemiology of multiple infections. *Trans. R. Soc. Trop. Med. Hyg.* **93** (Suppl 1), 59–64. 10.1016/s0035-9203(99)90329-2 (1999). 10450428 10.1016/s0035-9203(99)90329-2

[10] Essuman, E. et al. A novel gametocyte biomarker for superior molecular detection of the *Plasmodium falciparum* infectious reservoirs. *J. Infect. Dis.* **216**, 1264–1272. 10.1093/infdis/jix442 (2017). 28968664 10.1093/infdis/jix442

[11] Githeko, A. K. et al. The reservoir of *Plasmodium falciparum* malaria in a holoendemic area of western Kenya. *Trans. R. Soc. Trop. Med. Hyg.* **86**, 355–358. 10.1016/0035-9203(92)90216-y (1992). 1359683 10.1016/0035-9203(92)90216-y

[12] Lindblade, K. A., Steinhardt, L., Samuels, A., Kachur, S. P. & Slutsker, L. The silent threat: Asymptomatic parasitemia and malaria transmission. *Expert Rev. Anti Infect. Ther.* **11**, 623–639. 10.1586/eri.13.45 (2013). 23750733 10.1586/eri.13.45

[13] Okell, L. C. et al. Factors determining the occurrence of submicroscopic malaria infections and their relevance for control. *Nat. Commun.* **3**, 1237. 10.1038/ncomms2241 (2012). 23212366 10.1038/ncomms2241 PMC3535331

[14] Bousema, J. T. et al. *Plasmodium falciparum* gametocyte carriage in asymptomatic children in western Kenya. *Malar. J.* **3**, 18. 10.1186/1475-2875-3-18 (2004). 15202944 10.1186/1475-2875-3-18 PMC441400

[15] Koepfli, C. et al. Blood-stage parasitaemia and age determine *Plasmodium falciparum* and *P. vivax* gametocytaemia in Papua New Guinea. *PLoS One*. 10.1371/journal.pone.0126747 (2015). 10.1371/journal.pone.0126747 PMC4440770 25996916

[16] Mwingira, F., Genton, B., Kabanywanyi, A. N. M. & Felger, I. Comparison of detection methods to estimate asexual *Plasmodium falciparum* parasite prevalence and gametocyte carriage in a community survey in Tanzania. *Malar. J.* **13**, 433. 10.1186/1475-2875-13-433 (2014). 25404207 10.1186/1475-2875-13-433 PMC4246543

[17] Ouédraogo, A. L. et al. Dynamics of the human infectious reservoir for malaria determined by mosquito feeding assays and ultrasensitive malaria diagnosis in Burkina Faso. *J. Infect. Dis.* **213**, 90–99. 10.1093/infdis/jiv370 (2016). 26142435 10.1093/infdis/jiv370

[18] Ahmad, A. et al. Gametocyte carriage after seasonal malaria chemoprevention in *Plasmodium falciparum* infected asymptomatic children. *Malar. J.*10.21203/rs.3.rs-110518/v1 (2020). 33771166 10.1186/s12936-021-03706-1 PMC7995796

[19] Amoah, L. E., Abagna, H. B., Ayanful-Torgby, R., Blankson, S. O. & Aryee, N. A. Diversity and immune responses against *Plasmodium falciparum* gametocytes in non-febrile school children living in Southern Ghana. *Malar. J.* **18**, 265. 10.1186/s12936-019-2895-7 (2019). 31370841 10.1186/s12936-019-2895-7 PMC6676606

[20] Ouédraogo, A. L. et al. Substantial contribution of submicroscopical *Plasmodium falciparum* gametocyte carriage to the infectious reservoir in an area of seasonal transmission. *PLoS One* **4**, e8410. 10.1371/journal.pone.0008410 (2009). 20027314 10.1371/journal.pone.0008410 PMC2793432

[21] Mendis, K. N., Munesinghe, Y. D., de Silva, Y. N., Keragalla, I. & Carter, R. Malaria transmission-blocking immunity induced by natural infections of *Plasmodium vivax* in humans. *Infect. Immun.* **55**, 369–372. 10.1128/iai.55.2.369-372.1987 (1987). 2879793 10.1128/iai.55.2.369-372.1987 PMC260336

[22] Kumar, N. & Carter, R. Biosynthesis of the target antigens of antibodies blocking transmission of *Plasmodium falciparum*. *Mol. Biochem. Parasitol.* **13**, 333–342. 10.1016/0166-6851(84)90124-5 (1984). 6396517 10.1016/0166-6851(84)90124-5

[23] Jones, S. et al. Naturally acquired antibody responses to recombinant Pfs230 and Pfs48/45 transmission blocking vaccine candidates. *J. Infect.* **71**, 117–127. 10.1016/j.jinf.2015.03.007 (2015). 25869538 10.1016/j.jinf.2015.03.007

[24] Bousema et al. Human immune responses that reduce the transmission of *Plasmodium falciparum* in African populations. *Int. J. Parasitol.* **41**, 293–300. 10.1016/j.ijpara.2010.09.008 (2011). 20974145 10.1016/j.ijpara.2010.09.008 PMC3052432

[25] Bousema, Drakeley, C. J. & Sauerwein, R. W. Sexual-stage antibody responses to *P. falciparum* in endemic populations. *Curr. Mol. Med.* **6**, 223–229. 10.2174/156652406776055140 (2006). 16515512 10.2174/156652406776055140

[26] Healer, J. et al. Complement-mediated lysis of *Plasmodium falciparum* gametes by malaria-immune human sera is associated with antibodies to the gamete surface antigen Pfs230. *Infect. Immun.* **65**, 3017–3023 (1997). 9234748 10.1128/iai.65.8.3017-3023.1997 PMC175425

[27] McKenzie, F. E., Jeffery, G. M. & Collins, W. E. Plasmodium malariae infection boosts *Plasmodium falciparum* gametocyte production. *Am. J. Trop. Med. Hyg.* **67**, 411–414. 10.4269/ajtmh.2002.67.411 (2002). 12452496 10.4269/ajtmh.2002.67.411 PMC2504329

[28] Bruce, M. C. et al. Effect of transmission setting and mixed species infections on clinical measures of malaria in Malawi. *PLoS One* **3**, e2775. 10.1371/journal.pone.0002775 (2008). 18648666 10.1371/journal.pone.0002775 PMC2467490

[29] Mason, D. P. & McKenzie, F. E. Blood-stage dynamics and clinical implications of mixed *Plasmodium vivax*-*Plasmodium falciparum* infections. *Am. J. Trop. Med. Hyg.* **61**, 367–374. 10.4269/ajtmh.1999.61.367 (1999). 10497972 10.4269/ajtmh.1999.61.367 PMC2483693

[30] Obare, P. et al. Misclassification of Plasmodium infections by conventional microscopy and the impact of remedial training on the proficiency of laboratory technicians in species identification. *Malar. J.* **12**, 113. 10.1186/1475-2875-12-113 (2013). 23537145 10.1186/1475-2875-12-113 PMC3626703

[31] Bousema et al. Increased *Plasmodium falciparum* gametocyte production in mixed infections with *P. malariae*. *Am. J. Trop. Med. Hyg.* **78**(3), 442–448 (2008). 18337341

[32] Arez, A. et al. Transmission of mixed malaria species and strains by mosquitoes, as detected by PCR, in a study area in Guinea-Bissau. *Parassitologia* **59**, 65–70 (1997). 9419850

[33] Ouédraogo et al. A protocol for membrane feeding assays to determine the infectiousness of *P. falciparum* naturally infected individuals to *Anopheles gambiae*. *Malaria World Journal* **4** (2013). 10.5281/zenodo.10926272 PMC11138739 38828116

[34] McCaffery, J. N. et al. A multi-stage *Plasmodium vivax* malaria vaccine candidate able to induce long-lived antibody responses against blood stage parasites and robust transmission-blocking activity. *Front. Cell. Infect. Microbiol.*10.3389/fcimb.2019.00135 (2019). 10.3389/fcimb.2019.00135 PMC6504793 31119106

[35] Miura, K. et al. Malaria transmission-blocking vaccines: Wheat germ cell-free technology can accelerate vaccine development. *Expert Rev. Vaccines* **18**, 1017–1027. 10.1080/14760584.2019.1674145 (2019). 31566026 10.1080/14760584.2019.1674145 PMC11000147

[36] Ouédraogo, A. L. et al. Modeling the impact of *Plasmodium falciparum* sexual stage immunity on the composition and dynamics of the human infectious reservoir for malaria in natural settings. *PLoS Pathog.* **14**, e1007034. 10.1371/journal.ppat.1007034 (2018). 29742161 10.1371/journal.ppat.1007034 PMC5962096

[37] Stone, W. J. R. et al. Unravelling the immune signature of *Plasmodium falciparum* transmission-reducing immunity. *Nat. Commun.* **9**, 558–558. 10.1038/s41467-017-02646-2 (2018). 29422648 10.1038/s41467-017-02646-2 PMC5805765

[38] Amoah, L. E. et al. Dynamics of anti-MSP3 and Pfs230 antibody responses and multiplicity of infection in asymptomatic children from southern Ghana. *Parasites Vectors*. **11**, 13. 10.1186/s13071-017-2607-5 (2018). 29304870 10.1186/s13071-017-2607-5 PMC5755320

[39] Boudin, C., Olivier, M., Molez, J. F., Chiron, J. P. & Ambroise-Thomas, P. High human malarial infectivity to laboratory-bred *Anopheles gambiae* in a village in Burkina Faso. *Am. J. Trop. Med. Hyg.* **48**, 700–706. 10.4269/ajtmh.1993.48.700 (1993). 8517489 10.4269/ajtmh.1993.48.700

[40] Schneider, P. et al. Submicroscopic *Plasmodium falciparum* gametocyte densities frequently result in mosquito infection. *Am. J. Trop. Med. Hyg.* **76**, 470–474 (2007). 17360869

[41] Beshir, K. B. et al. Residual *Plasmodium falciparum* parasitemia in Kenyan children after artemisinin-combination therapy is associated with increased transmission to mosquitoes and parasite recurrence. *J. Infect. Dis.*10.1093/infdis/jit431 (2013). 23945376 10.1093/infdis/jit431 PMC3836468

[42] Vantaux, A. et al. Contribution to malaria transmission of symptomatic and asymptomatic parasite carriers in Cambodia. *J. Infect. Dis.* **217**, 1561–1568. 10.1093/infdis/jiy060 (2018). 29394367 10.1093/infdis/jiy060

[43] Graves, P. M. et al. Naturally occurring antibodies to an epitope on *Plasmodium falciparum* gametes detected by monoclonal antibody-based competitive enzyme-linked immunosorbent assay. *Infect. Immun.* **56**, 2818–2821. 10.1128/IAI.56.11.2818-2821.1988 (1988). 2459062 10.1128/iai.56.11.2818-2821.1988 PMC259655

[44] Gebru, T. et al. Recognition of *Plasmodium falciparum* mature gametocyte-infected erythrocytes by antibodies of semi-immune adults and malaria-exposed children from Gabon. *Malar. J.* **16**, 176. 10.1186/s12936-017-1827-7 (2017). 28446190 10.1186/s12936-017-1827-7 PMC5406886

[45] van Schaijk, B. C. L. et al. Pfs47, paralog of the male fertility factor Pfs48/45, is a female specific surface protein in *Plasmodium falciparum*. *Mol. Biochem. Parasitol.* **149**, 216–222. 10.1016/j.molbiopara.2006.05.015 (2006). 16824624 10.1016/j.molbiopara.2006.05.015

[46] Lensen, A., Bolmer-Van de Vegte, M., Van Gemert, G., Eling, W. & Sauerwein, R. Leukocytes in a *Plasmodium falciparum*-infected blood meal reduce transmission of malaria to Anopheles mosquitoes. *Infect. Immun.* **65**, 3834–3837 (1997). 9284160 10.1128/iai.65.9.3834-3837.1997 PMC175547

[47] Muniz-Junqueira, M. I., dos Santos-Neto, L. L. & Tosta, C. E. Influence of tumor necrosis factor-α on the ability of monocytes and lymphocytes to destroy intraerythrocytic *Plasmodium falciparum* in vitro. *Cell. Immunol.* **208**, 73–79 (2001). 11333139 10.1006/cimm.2001.1770

[48] Sinden & Smalley, M. Gametocytes of *Plasmodium falciparum*: Phagocytosis by leucocytes in vivo and in vitro. *Trans. R. Soc. Trop. Med. Hyg.* **70**, 344–345. 10.1016/0035-9203(76)90096-1 (1976). 795106 10.1016/0035-9203(76)90096-1

[49] Zhang et al. Anopheles midgut FREP1 mediates Plasmodium invasion. *J. Biol. Chem.* **290**, 16490–16501. 10.1074/jbc.M114.623165 (2015). 25991725 10.1074/jbc.M114.623165 PMC4505404

[50] Eldering, M. et al. Variation in susceptibility of African *Plasmodium falciparum* malaria parasites to TEP1 mediated killing in *Anopheles gambiae* mosquitoes. *Sci. Rep.* **6**, 20440–20440. 10.1038/srep20440 (2016). 26861587 10.1038/srep20440 PMC4748223

[51] Bousema & Drakeley, C. Epidemiology and infectivity of *Plasmodium falciparum* and *Plasmodium vivax* gametocytes in relation to malaria control and elimination. *Clin. Microbiol. Rev.* **24**, 377–410. 10.1128/CMR.00051-10 (2011). 21482730 10.1128/CMR.00051-10 PMC3122489

[52] Kumar, N. Target antigens of malaria transmission blocking immunity exist as a stable membrane bound complex. *Parasite Immunol.* **9**, 321–335. 10.1111/j.1365-3024.1987.tb00511.x (1987). 3299225 10.1111/j.1365-3024.1987.tb00511.x

[53] van der Kolk, M., de Vlas, S. J. & Sauerwein, R. W. Reduction and enhancement of *Plasmodium falciparum* transmission by endemic human sera. *Int. J. Parasitol.* **36**, 1091–1095. 10.1016/j.ijpara.2006.05.004 (2006). 16790244 10.1016/j.ijpara.2006.05.004

[54] Acquah, F. K. et al. Asymptomatic carriage of *Plasmodium falciparum* by individuals with variant blood groups and haemoglobin genotypes in southern Ghana. *Malar. J.* **19**, 217. 10.1186/s12936-020-03299-1 (2020). 32576186 10.1186/s12936-020-03299-1 PMC7310487

[55] Diallo, M. et al. Evaluation and optimization of membrane feeding compared to direct feeding as an assay for infectivity. *Malar. J.* **7**, 248. 10.1186/1475-2875-7-248 (2008). 19055715 10.1186/1475-2875-7-248 PMC2640402

[56] Das, Garver, L. & Dimopoulos, G. Protocol for mosquito rearing (A. gambiae). *J. Vis. Exp.*. 10.3791/221 (2007). 10.3791/221 PMC2557088 18979019

[57] Singh, B. et al. A genus- and species-specific nested polymerase chain reaction malaria detection assay for epidemiologic studies. *Am. J. Trop. Med. Hyg.* **60**, 687–692. 10.4269/ajtmh.1999.60.687 (1999). 10348249 10.4269/ajtmh.1999.60.687

[58] Menegon, M. et al. Genotyping of *Plasmodium falciparum* gametocytes by reverse transcriptase polymerase chain reaction. *Mol. Biochem. Parasitol.* **111**, 153–161. 10.1016/S0166-6851(00)00314-5 (2000). 11087925 10.1016/s0166-6851(00)00314-5

[59] Acquah, F. K. et al. Antibody responses to two new Lactococcus lactis-produced recombinant Pfs48/45 and Pfs230 proteins increase with age in malaria patients living in the Central Region of Ghana. *Malar. J.* **16**, 306. 10.1186/s12936-017-1955-0 (2017). 28764709 10.1186/s12936-017-1955-0 PMC5540549

