# Supplementary figures and images for: Assessment of the infectivity of malaria parasites from asymptomatic school children to Anopheles gambiae mosquitoes in a high transmission area in Ghana
Source: Sci Rep. 2025 Jul 2;15:22561. doi: 10.1038/s41598-025-06844-7 (PMC12219327; doi:10.1038/s41598-025-06844-7)

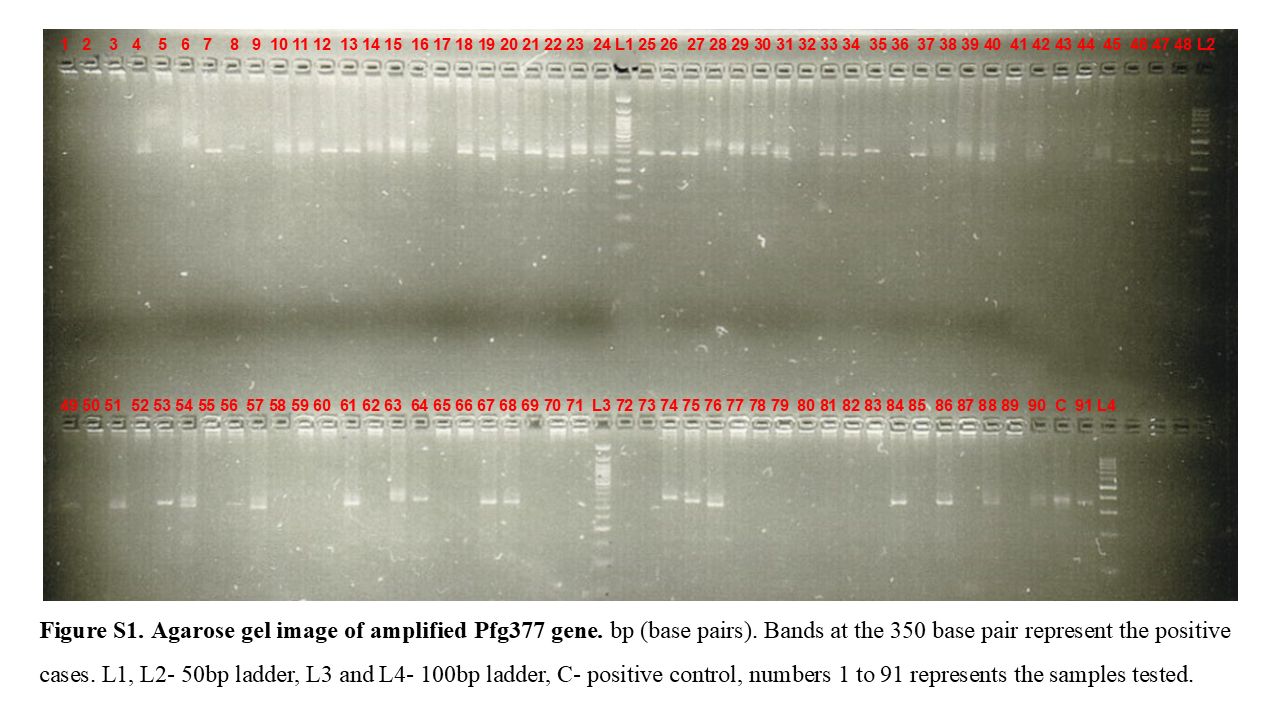

Supplement: Supplementary file 1 — Supplementary Material 1 [file 41598_2025_6844_MOESM1_ESM.tif]
